# Colorectal Cancer: Differential Gene Expression and In Vitro Response to 5-Fluorouracil, Novel Fluoropyrimidine F10, and Potential Synergy with Lupeol

**DOI:** 10.3390/ijms262211134

**Published:** 2025-11-18

**Authors:** Shrey D. Thaker, Jenny Paredes, Jone Garai, Laura A. Martello, William H. Gmeiner, Jovanny Zabaleta, Jennie Williams

**Affiliations:** 1 Department of Family, Population, Preventative Medicine, Stony Brook University, Stony Brook, NY 11794, USA; shrey.thaker@stonybrookmedicine.edu; 2 Division of Gastroenterology & Hepatology, SUNY Downstate Health Sciences University, Brooklyn, New York City, NY 11203, USA; 3 Stanley S. Scott Cancer Center, LSUHSC-New Orleans, New Orleans, LA 70112, USA; 4 Department of Cancer Biology, Wake Forrest School of Medicine, Winston-Salem, NC 27109, USA; 5 Department of Interdisciplinary Oncology, LSUHSC-New Orleans, New Orleans, LA 70112, USA

**Keywords:** racial health disparity, colorectal cancer, fluoropyrimidine, 5-fluorouracil, lupeol

## Abstract

Colorectal cancer (CRC) remains one of the most lethal malignancies in the United States, with African American (AA) patients experiencing disproportionately higher incidence and mortality compared to Caucasian Americans (CAs). These disparities have been linked to tumor-intrinsic genomic differences, including microsatellite instability (MSI) and p53 mutation status, which may influence chemotherapeutic response, particularly to the standard-of-care agent 5-fluorouracil (5-FU). However, mechanistic insights have been limited by the lack of racially diverse preclinical models. Here, we evaluated the efficacy of F10 (a novel fluoropyrimidine polymer) vs. 5-FU using AA- and CA-derived CRC cell lines with distinct MSI and p53 profiles. MTT assays revealed that MSI status, more than racial origin, predicted 5-FU sensitivity. Transcriptomics uncovered distinct gene expression patterns associated with MSI status and racial background, particularly in drug metabolism pathways. F10 demonstrated superior potency and consistency vs. 5-FU across all cell lines, independent of race, MSI, or p53 status. Additionally, in silico docking and immunofluorescence suggest that the dietary triterpene lupeol enhances F10 efficacy, perhaps through stabilization of the Fas apoptosis pathway. These findings underscore the therapeutic potential of F10 and the importance of integrating diverse tumor models with dietary adjuvants to inform more effective and inclusive CRC treatment strategies.

## 1. Introduction

Colorectal cancer (CRC) is the third leading cause of cancer-related deaths in the United States, claiming approximately 50,000 lives annually. African American (AA) patients experience a 30–50% higher mortality rate compared to Caucasian American (CA) patients, even when controlling for socioeconomic and environmental factors [1,2,3,4]. Despite improvements in screening and early detection, this disparity in CRC outcomes persists [5,6,7,8,9,10,11,12], suggesting that tumor-intrinsic biological differences may contribute to differential treatment responses. Recent studies have identified biomolecular distinctions between AA and CA CRC tumors that may underlie these disparities. AA tumors exhibit a higher frequency of single nucleotide polymorphisms (SNPs) in the tumor suppressor gene *TP53* [13,14,15], differential CpG island methylation patterns [16,17,18], and altered microRNA (miRNA) profile expression [19,20]. These molecular differences may influence tumorigenesis, progression, and response to chemotherapy.

Fluoropyrimidine-based chemotherapy, particularly 5-fluorouracil (5-FU), remains a cornerstone of CRC treatment. However, AA patients show significantly differential response to 5-FU compared to CA patients [21,22,23,24]. Mismatch repair (MMR) proteins, which regulate genomic stability and apoptosis in response to fluoropyrimidines, vary between AA and CA tumors [25,26,27,28,29,30,31,32]. Approximately 15% of CRC tumors exhibit high microsatellite instability (MSI), a marker of deficient MMR, which is associated with reduced sensitivity to 5-FU [26,33,34,35,36,37]. Moreover, p53 mutations inversely correlate with MSI status and are implicated in fluoropyrimidine resistance [38,39,40,41,42]. These findings suggest that MSI and p53 status may be key predictors of chemotherapy response and contributors to racial disparities in CRC outcomes. Historically, the lack of AA-derived CRC cell lines has limited mechanistic studies of racial disparities in drug response. To address this gap, we incorporated in this study three AA CRC cell lines (SB501, SB521, and CHTN06) characterized to have distinct MSI and p53 profiles [43]. SB501 is MSI-L, SB521 is MSI-H, and CHTN06 is MSS. These cell lines provide a unique opportunity to investigate fluoropyrimidine response in a racially inclusive, tumor-intrinsic context.

While 5-FU has been used for over 50 years and treats millions of cancer patients annually [44,45,46,47,48,49,50], its efficacy is limited by systemic toxicity and variable tumor sensitivity [51,52,53,54,55,56,57]. F10, a polymer of FdUMP (the active metabolite of 5-FU), was developed to overcome these limitations [58]. F10 exhibits enhanced potency through efficient conversion to FdUMP and dual targeting of thymidylate synthase (TYMS) and DNA topoisomerase I [59,60,61,62]. It selectively targets rapidly dividing cells, reducing off-target toxicity [63], and has shown promise in leukemia and other cancer models [64,65,66]. In some of these models, F10 activates the Fas-mediated extrinsic apoptosis pathway [66], which, compared to the intrinsic pathway, offers advantages such as p53-independence and fewer mutational bottlenecks [67,68]. If shown to be relevant in CRC, therapeutics that leverage Fas pathway activation may be further potentiated by other compounds known to enhance Fas signaling. Recently, dietary management strategies to enhance chemotherapy efficacy have been emerging [69,70]. From the perspective of Fas pathway activation, the dietary micronutrient lupeol, a triterpene found in Mediterranean fruits, has been shown to stabilize lipid rafts and promote Fas receptor trimerization, enhancing extrinsic apoptosis [71,72,73,74,75,76].

This study aims to (1) characterize the response of AA CRC cell lines to 5-FU and F10, (2) assess the predictive power of MSI and p53 status in fluoropyrimidine sensitivity, (3) investigate transcriptomic differences between AA and CRC cell lines, and (4) explore the potential synergy between F10 and lupeol via the Fas-mediated apoptosis pathway. We hypothesize that F10 will be more efficacious than 5-FU, but we expect that its relative improvement will be associated with tumor-intrinsic characteristics observed in AA and CA CRC tumors. We further hypothesize that lupeol may synergize with F10 to further potentiate its efficacy in CRC. By integrating novel AA CRC models, a next-generation chemotherapeutic, and a dietary adjuvant, this study seeks to advance personalized medicine and reduce divergences in CRC treatment outcomes.

## 2. Results

### 2.1. MSI Is a Strong Predictor of 5-FU Efficacy In Vitro

**Figure 1:**
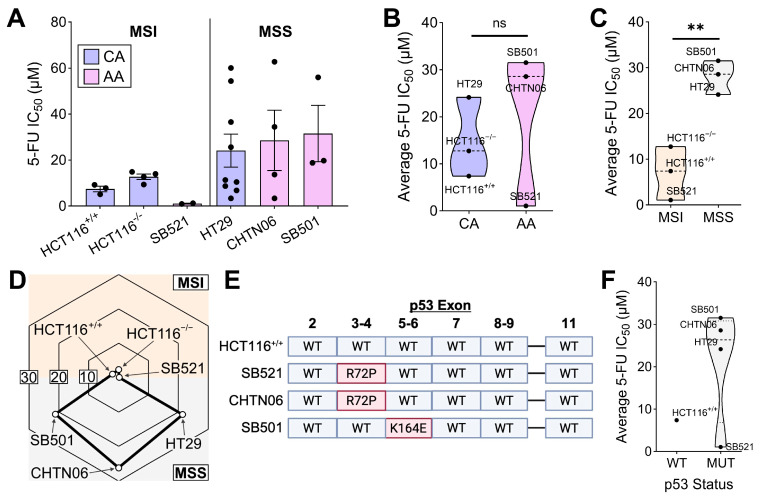
MSI status is a strong predictor of 5-FU sensitivity in both CA and AA CRC cell lines. (**A**) Indicated cell lines were treated with 5-FU for 72 h and MTT assay was performed. IC_50_ values for determined using 4−parameter regression, with each data point representing the IC_50_ value from an independent trial for each cell line (*n* = 3 to 9 replicate experiments per cell line). (**B**,**C**) Average 5-FU IC_50_ value for each cell line plotted by racial origin (**B**) and MSI status (**C**). (**D**) The standard deviation of 5-FU IC_50_ values across all independent trials for each cell line was plotted on a radar chart, with higher values on the periphery and lower values in the center. (**E**) The exons of p53 of the indicated cell lines were sequenced with Sanger sequencing, with mutation(s) displayed in red (Created in *BioRender. Thaker, S. (2025)* https://BioRender.com/wfs02yw). (**F**) Average 5-FU IC_50_ value for each cell line plotted by p53 status. ** *p* < 0.01.

Given the epidemiological connections between racial background and 5-FU treatment outcomes [21,22,24], we first asked whether these associations were tumor-intrinsic. We leveraged our previously reported primary colorectal cancer (CRC) cell lines derived from patients of African American (AA) origin [43]. As a comparator, we used commercially available Caucasian American (CA) CRC cell lines, HCT116 and HT29. Lastly, given the central role of p53 in tumor response to fluoropyrimidines [39,77], we also tested HCT116^+/+^ (WT-p53) and HCT116^−/−^ (KO-p53). Each of these cell lines were treated with 5-FU for 72 h followed by the MTT metabolic viability assay as a readout of 5-FU response. 5-FU exhibited the lowest IC_50_ value in the AA cell line SB521 followed by the CA cell line HCT116^+/+^, whereas the AA cell lines SB501 and CTHN06 exhibited the highest average IC_50_ values (Figure 1A). Notably, while racial origin was initially hypothesized to be a strong predictor of in vitro 5-FU response, MSI status emerged as a stronger correlation (Figure 1B,C). Another clinical concern is patient-to-patient and within-patient inconsistency in response to fluoropyrimidines [78,79,80,81]. Thus, we assessed the variation in IC_50_ value of 5-FU across independent trials for each cell line, made possible by all cell lines being tested within each trial contemporaneously with the agent to minimize technical variation. This analysis revealed extensive variation only in MSS, but not MSI, cell lines (Figure 1D). Finally, because p53 status is centrally related to fluoropyrimidine response [39,82], we sequenced the exons of p53 in each cell line, revealing that all our AA CRC cell line models harbor mutant p53 with varying biological consequences (Figure 1E). In addition to MSI status, p53 mutant status appears to be a strong negative predictor of 5-FU sensitivity in our model, except in the case of SB521 (Figure 1F). However, further wild-type comparators are needed to properly interpret this association. Altogether, these data suggest that (1) our in vitro AA CRC cell line models reflect clinical correlations between tumor biomarkers and fluoropyrimidine response, and (2) that tumor cell-intrinsic factors are sufficient to impact 5-FU sensitivity.

### 2.2. MSI and Racial Origin Are Associated with Unique Transcriptomes

**Figure 2:**
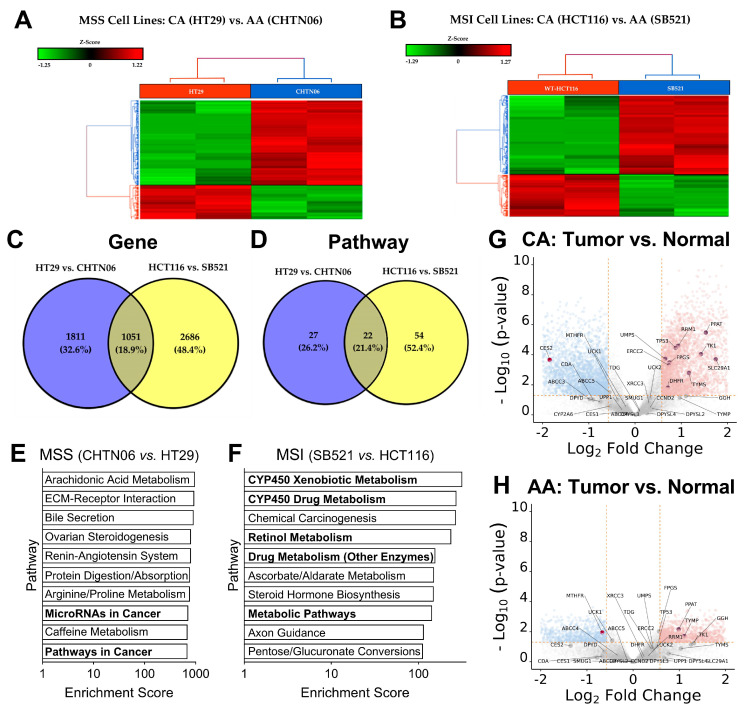
MSI and racial origin have a layered impact on CRC transcriptomes, specifically on drug metabolism pathways. (**A**,**B**), Extracted RNA from indicated cell lines was analyzed with bulk RNA sequencing. Unsupervised hierarchical clustering of gene expression patterns was conducted for MSS cell lines (**A**) and MSI cell lines (**B**). (**C**,**D**), Layered analysis of gene expression made relative to respective AA cell line compared between MSI status at the individual gene level (**C**) and pathway enrichment level (**D**). (**E**,**F**), Pathway enrichment analysis showing top 10 differentially expressed pathways between AA and CA cell line within the MSS group (**E**) and the MSI group (**F**). (**G**,**H**), Gene expression of fluoropyrimidine metabolism pathway analyzed from our previously published RNAseq dataset [83] comparing tumor vs. healthy adjacent tissue gene expression from CA (*n* = 20) (**G**) and AA (*n* = 20) (**H**) CRC tumors. Light blue dots are significantly downregulated genes, light red dots are significantly upregulated genes, and dark red dots with labels are genes belonging to the fluoropyrimidine metabolism pathway.

To gain deeper insight into the biomolecular mechanisms influencing 5-FU sensitivity beyond static biomarkers such as MSI status and racial origin, we performed bulk RNA sequencing on our CRC cell lines. Given the established relevance of these two variables in fluoropyrimidine response, we compared the gene expression profiles of (1) an AA MSS cell line (CHTN06) to a CA MSS cell line (HT29) and (2) an AA MSI cell line (SB521) to a CA MSI cell line (HCT116^+/+^). Within each microsatellite status group (MSS and MSI), the AA and CA cell lines clustered separately, indicating that racial origin is associated with distinct transcriptional profiles (Figure 2A,B). To determine whether these race-associated transcriptomic differences were influenced by MSI status, we compared each AA cell line to its MSI-matched CA counterpart (i.e., CHTN06 to HT29 and SB521 to HCT116). Differential expression analysis revealed that both gene-level and pathway-level profiles clustered distinctly between AA and CA cell lines, regardless of MSI status (Figure 2C,D). Importantly, pathway enrichment analysis showed that the differentially expressed pathways varied between the MSS and MSI comparison groups (Figure 2E,F), supporting the conclusion that racial origin and MSI status independently contribute to transcriptomic variation. These findings underscore the complexity of tumor-intrinsic biology and suggest that both race and MSI status may shape the molecular landscape relevant to chemotherapeutic response.

Given the differences in drug-related metabolic pathways between the MSI cell lines SB521 and HCT116 (Figure 2F), we next investigated whether differential expression of the fluoropyrimidine metabolism pathway was correlated with these cell lines’ divergent response to 5-FU. Analysis of our previously published RNAseq dataset [83], which compared CA and AA CRC tumors normalized to healthy adjacent tissue, revealed that CA tumors exhibited significantly greater upregulation of fluoropyrimidine pathway genes [84] vs. healthy adjacent tissue (Figure 2G) compared to AA tumors (Figure 2H). This led us to hypothesize that similar differences might be reflected in our in vitro models and correlate with 5-FU sensitivity. However, among the 34 genes in the fluoropyrimidine metabolism pathway, only two genes were significantly different between SB521 and HCT116, and only one gene between CHTN06 and HT29. This suggests that a factor beyond gene expression of this pathway—such as post-transcriptional regulation, DNA repair capacity, apoptotic signaling, or alternative pathways—may have a greater impact on 5-FU response in these cell lines. For example, SB521 exhibited elevated expression of carcinoembryonic antigen (CEA), a biomarker previously linked to enhanced 5-FU sensitivity [85,86], which may partially explain its heightened responsiveness. While our cell line data did not recapitulate the full extent of fluoropyrimidine pathway expression differences observed at the gene expression level in tumor tissue, the tumor dataset supports the hypothesis that differential 5-FU metabolism may contribute to racial disparities in CRC 5-FU treatment outcomes.

### 2.3. The Novel Fluoropyrimidine F10 Overcomes Negative Predictors of 5-FU Sensitivity

**Figure 3:**
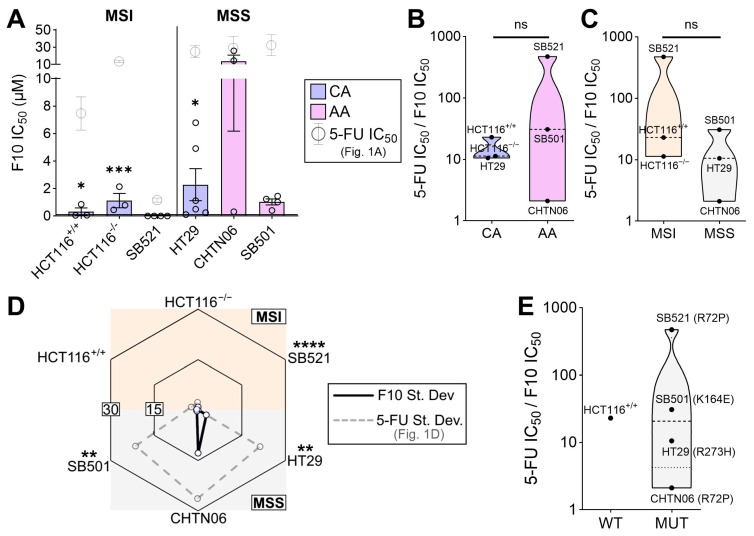
F10 demonstrates enhanced efficacy in vitro regardless of negative predictors of fluoropyrimidine response. (**A**) Indicated cell lines were treated with 5-FU for 72 h and MTT assay was performed. IC_50_ values for determined using 4-parameter regression, with each data point representing an independent trial for that cell line (*n* = 3 to 6 independent experiments per cell line). Statistical significance compares the 5-FU IC_50_ values from Figure 1A (indicated in gray) to those of F10 for the same cell line. Error bars represent SEM. Both drugs were tested contemporaneously in the same experiment, allowing for such comparison. (**B**,**C**) The relative efficacy of F10 was calculated by dividing 5-FU IC_50_ by F10 IC_50_, whereby higher values indicate greater F10 efficacy vs. 5-FU, stratified by racial origin (**B**) and MSI status (**C**). (**D**) The standard deviation of F10 IC_50_ values for each cell line was plotted on the radar chart, with higher values on the periphery and lower values in the center. Gray data points are 5-FU IC_50_ standard deviations from Figure 1D, and statistical indicators are F-tests comparing variance of 5-FU and F10 for each cell line. (**E**) Relative F10 efficacy for each cell line plotted by p53 status. * *p* < 0.05, ** *p* < 0.01, *** *p* < 0.001, **** *p* < 0.0001.

Our findings thus far suggest that tumor-intrinsic properties (e.g., MSI status) correlate with 5-FU sensitivity, reflecting a significant clinical challenge. Therefore, we next asked whether the novel, next-generation fluoropyrimidine F10 [63] can remain effective in MSS and AA cell lines which were less sensitive and varied extensively in response to 5-FU (Figure 1A,D). Strikingly, F10 significantly outperformed 5-FU in all cell lines tested (Figure 3A). To better capture the magnitude by which F10 was more effective compared to 5-FU, the IC_50_ of 5-FU was divided by that of F10. Larger numbers indicate a greater efficacy of F10 relative to 5-FU. This relative F10 efficacy did not correlate with racial origin nor with MSI status (Figure 3B,C), and the magnitude for all cell lines was above 1, which indicates that regardless of these biomarkers, F10 was more effective than 5-FU at reducing metabolic viability. Further, F10 was significantly more consistent across independent trials compared to 5-FU, especially in cell lines that previously had a large standard deviation across trials in response to 5-FU (Figure 3D). Given that the 5-FU and F10 experiments were done synchronously on each cell line, the difference in trial-to-trial variation likely reflects a true biological (rather than technical) variation. Lastly, the relative F10 efficacy was stratified by p53 status, and while no correlation between WT and mutant p53 can be made, all cell lines with mutant p53 exhibit a relative F10 efficacy greater than 1, indicating that F10 is more effective than 5-FU despite mutant p53 (Figure 3E). These results suggest that F10, a next-generation fluoropyrimidine, is a promising candidate which may equalize the therapeutic opportunity for difficult-to-treat tumors.

### 2.4. Synergy of F10 FAS-Mediated Apoptosis and Dietary Lupeol

**Figure 4:**
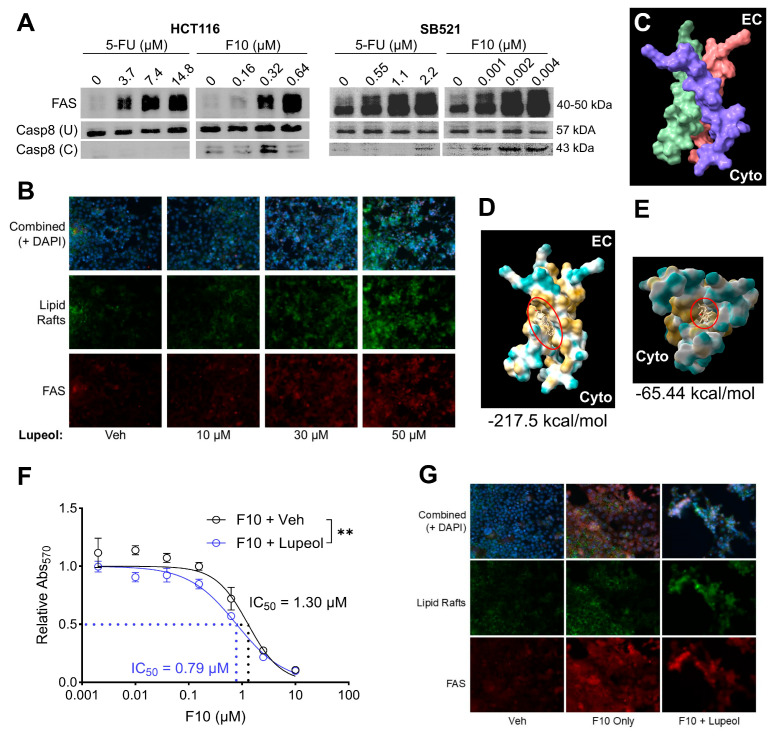
Lupeol may synergize with F10 by potentiating FAS receptor stabilization. (**A**) HCT116 and SB521 were treated with 5-FU or F10 for 72 h at indicated concentrations. FAS receptor expression and Caspase 8 cleavage (U = uncleaved, C = cleaved) was assessed by Western Blot, reproduced twice. (**B**) HCT116 treated with indicated concentrations of lupeol for 72 h with lipid rafts and FAS receptor expression assessed by immunofluorescence. (**C**) Previously published trimerized transmembrane domains of FAS receptor [87] (EC = extracellular side, Cyto = cytoplasmic side, each monomer is a different color). (**D**,**E**), In silico docking analysis between lupeol (red circle, beige chemical structure) and the FAS transmembrane trimer (yellow = hydrophobic, blue = hydrophilic) with most stable (**D**) and second-most stable (**E**) structures shown. Numbers below each image represent predicted binding energies (kcal/mol). (**F**) HCT116 treated with F10 + Veh or F10 + Lupeol for 72 h followed by MTT assay and 4–parameter regression analysis. Significance indicates F-test for IC_50_ difference. (**G**) HCT116 treated with no drug (Veh), F10 Only, or F10 + Lupeol for F10 with lipid rafts and FAS receptor expression by immunofluorescence. ** *p* < 0.01.

Given the promising efficacy of F10 in cell lines that were previously resistant to 5-FU, we sought a mechanism to further potentiate F10 efficacy. F10 has been reported to activate extrinsic, FAS-mediated apoptosis in other cancer models [66]. Thus, we first assessed if F10 impacts the FAS pathway in fluoropyrimidine-sensitive CRC cell lines, HCT116 and SB521. Here, we found a dose-dependent relationship between F10 treatment and FAS receptor expression, and F10 treatment induced cleavage of Caspase 8 (Casp8), the extrinsic initiator caspase (Figure 4A). While higher concentrations of 5-FU demonstrated a similar relationship with FAS receptor expression, there was minimal induction of Casp8 cleavage (Figure 4A), suggesting that extrinsic apoptosis is uniquely induced by F10 and not 5-FU. Because diet can impact CRC pathogenesis and response to chemotherapy [69,70], we next asked whether a dietary micronutrient can further enhance the already promising efficacy of F10 through the FAS pathway. FAS activation by F10 was associated with increased density of lipid rafts in the plasma membrane, wherein three FAS monomers can trimerize (and thus activate) more easily [66,67,87]. Lupeol—a dietary triterpene with antioxidant and antitumor properties found in tropical and Mediterranean fruits [74,75,76]—is highly hydrophobic, which we hypothesized could further influence lipid raft formation. Immunofluorescence staining after lupeol treatment of HCT116 revealed a dose-dependent increase in lipid raft density (Figure 4B). This increase was also associated with greater colocalized FAS receptor expression (Figure 4B). Due to its hydrophobic nature and the affinity of FAS trimers to lipid rafts, we further posited that lupeol may directly stabilize the hydrophobic transmembrane domains of the FAS trimer. Indeed, in silico docking analysis revealed two distinct binding pockets for lupeol within the FAS trimer (Figure 4C–E), both of which could substantially enhance hydrophobic interactions and enhance FAS trimer formation. Thus, in our CRC models, we see evidence of F10-induced FAS pathway activation, and stabilization of the active form of FAS receptor and supportive membrane architecture by lupeol.

To test if these parallel mechanisms of F10 and lupeol could synergize functionally, we assessed if co-treatment of HCT116 with these two compounds would impact F10 IC_50_ by MTT assay. Indeed, lupeol treatment increased the sensitivity to F10, reducing the IC_50_ by about 40% from 1.30 μM to 0.79 μM (Figure 4F). Additionally, lupeol supplementation increased the presence of apoptotic morphology at the same dose of F10 compared to vehicle (Figure 4G). Altogether, these findings suggest that F10, while effective on its own compared to the standard of care 5-FU, may be further empowered by the presence of beneficial dietary micronutrients such as lupeol.

## 3. Discussion

The epidemiological association between race and CRC has been extensively studied from a socioeconomic perspective [88]. However, our study contributes to the growing body of evidence supporting the role of tumor-intrinsic biomolecular mechanisms that may further explain racial disparities in CRC outcomes. By leveraging our previously established in vitro models derived from African American (AA) patients [43], we systematically investigated the interplay between race, MSI, and chemotherapeutic response in a controlled experimental setting. Our findings support four key conclusions: (1) MSI status is a robust predictor of 5-FU sensitivity, independent of racial origin; (2) race and MSI status exert a layered influence on the transcriptional profiles of CRC cells; (3) the novel fluoropyrimidine F10 demonstrates superior potency and consistency compared to 5-FU across diverse cell profiles, including those with p53 mutations; and (4) the dietary triterpene lupeol may enhance F10 efficacy through co-activation of the Fas-mediated extrinsic apoptosis pathway.

Bulk RNA sequencing revealed distinct transcriptional landscapes between AA and CA CRC cell lines, even within matched MSI/MSS cohorts. MSI cell lines exhibited greater divergence in oncogenic and metabolic pathways, including upregulation of the carcinoembryonic antigen (CEA) family in AA MSI cell line SB521. Given that CEA overexpression has been linked to both metastatic potential and increased 5-FU sensitivity [85,86,89], this may partially explain SB521′s heightened drug responsiveness. Interestingly, while our tumor RNAseq dataset showed significant differences in fluoropyrimidine metabolism gene expression between AA and CA tumors relative to normal adjacent tissue, these differences were not recapitulated in the cell line models. This discrepancy underscores the complexity of translating tumor-level observations to in vitro systems and suggests that additional regulatory mechanisms, such as post-transcriptional control or epigenetic modulation, may influence the response to 5-FU. Ultimately, a more detailed pharmacokinetic profile of 5-FU and its downstream metabolites within each cell line would provide the necessary clarity on how its metabolism may functionally differ and perhaps influence each cell line’s response.

Our data robustly demonstrate that F10 is more potent and consistent than 5-FU in reducing CRC metabolic viability across all cell lines tested. This aligns with previous studies showing F10′s superior efficacy due to its polymeric structure, efficient conversion to FdUMP, and dual inhibition of thymidylate synthase and topoisomerase I across multiple cancers, including CRC [59,61,62,90,91,92,93]. Though 5-FU is known to be taken up rapidly by proliferating cells, it must be extensively metabolized into several toxic intermediates (such as the neurotoxic compound F-BAL [54]) before reaching the desired active compound FdUMP [84]. A significant challenge posed by such a mechanism is the capacity of these toxic metabolites to diffuse out of the target cell and induce systemic toxicity [54,55]. In contrast, F10, which is a modified polymer of the active compound FdUMP, is actively transported and metabolized via a simpler hydrolytic cleavage by cytosolic and nuclear exonucleases [59,60], reducing inter-sample variability and off-target toxicity observed with 5-FU. Importantly, F10 retained efficacy in cell lines harboring p53 mutations, a known barrier to 5-FU response [39,40,42,78], suggesting that F10 may engage alternative apoptotic pathways. This is supported by our data and prior studies indicating F10′s engagement of the p53-independent, Fas-mediated extrinsic apoptosis pathway [66].

Contrary to clinical expectations [35,36,37], our MSI cell lines were more sensitive to both 5-FU and F10 compared to the MSS cell lines. This observation may reflect the impaired DNA repair capacity of MSI cells, rendering them ill-equipped to recover from F10-induced double-stranded breaks via Top1 cleavage complexes [94,95,96]. Among all cell lines, SB521, an MSI AA cell line, exhibited the greatest sensitivity to both agents, further supporting the hypothesis that MSI status, rather than racial origin, is a dominant determinant of fluoropyrimidine response.

To further enhance F10′s therapeutic potential, we investigated the dietary compound lupeol [69,70,71,72,73], known to stabilize lipid rafts and promote Fas receptor trimerization [74,75,76]. While both 5-FU and F10 increased Fas receptor expression, only F10 induced caspase-8 cleavage, indicating selective activation of the extrinsic apoptotic pathway. Co-treatment with lupeol significantly reduced F10′s IC50 value and increased apoptotic morphology, suggesting a synergistic interaction. Our in silico docking analysis revealed two potentially favorable physical binding pockets for lupeol within the transmembrane domains of the Fas trimer, which supports the hypothesis that lupeol may physically stabilize the active trimer form of the Fas receptor, making cells more susceptible to Fas-targeting drugs. Indeed, a similar approach with a static binding algorithm has been conducted with lupeol in the context of intracellular tumor promoting proteins, such as mTOR, topoisomerase, Bcl-2, and PI3K [97]. Complementing the binding energies in that study, which ranged from −11.56 kcal/mol to −6.82 kcal/mol [97], our work adds credence to the potential antitumor biochemistry of lupeol with even more favorable predicted binding energies to the activated Fas trimer (−217.5 and −65.44 kcal/mol). These values suggest that lupeol may have greater stability when binding to the Fas trimer compared to the previously predicted intracellular oncoproteins, which may be reasonable given lupeol’s high hydrophobicity and the Fas trimer’s highly hydrophobic transmembrane domain as opposed to more polar intracellular targets. These findings provide a compelling rationale for future mechanistic studies including time-course studies and confocal microscopy to elucidate the kinetics of Fas pathway engagement by F10 and lupeol. Additionally, planned Fas receptor knockdown studies will further clarify its role in mediating the observed synergy between F10 and lupeol.

## 4. Materials and Methods

Cell Lines. CA CRC cell lines HT29, HCT116^+/+^ (wild-type p53), and HCT116^−/−^ (p53-null) were obtained commercially from ATCC (Manassas, VA, USA). These cells were cultured in McCoy’s 5a media (Corning Cellgro, New York, NY, USA) supplemented with 10% FBS, 100 U/mL penicillin and 100 µg/mL streptomycin. AA CRC cell lines SB501, SB521, and CHTN06 were generated in our lab as previously described [43]. Importantly, all experiments carried out in this study utilizing these AA cell lines were executed using liquid nitrogen-frozen stocks from the same batch of cells extensively characterized in our previous publication, which included their STR profiling, MSI profiling, and other authenticating verifications [43]. AA CRC cell lines were maintained in DMEM (Corning Cellgro, New York, NY, USA) supplemented with 10% FBS, 100 U/mL penicillin, 100 U/mL streptomycin, 15 μg/mL ciprofloxacin, 2.5 μg/mL amphotericin B, 50 μg/mL gentamycin, 0.25 ng/mL epidermal growth factor, 1% insulin, 5 mg/mL transferrin and 20 μg/mL selenium (all from Sigma-Aldrich, St. Louis, USA; Merck KGaA, Darmstadt, Germany). All cell lines were maintained at 37 °C in 5% CO_2_.

Drug Preparation. 5-FU stock solution was prepared fresh prior to all experiments in DMSO at 50 mg/mL. F10 stock solution was prepared in sterile phosphate-buffered saline (Corning Cellgro, New York, NY, USA) at 30 mM and stored at −20 °C. Because F10 is a 10-unit polymer of the fluorinated nucleotide, FdUMP, it was further possible to verify its concentration in solution using the ssDNA setting on the Nanodrop 2000 UV spectrophotometer (Thermo Fisher Scientific, Rockford, IL, USA) and using the known molecular weight of F10 (MW = 3009 g/mol) to convert the detected of F10 in ng/μL (by Nanodrop) to molar concentration. In contrast, 5-FU could not be verified this way due to its monomeric nature, so its preparation was conducted with the traditional powder mass and solvent volume methods. Both drug stock concentrations were well within their solubilities (thus no precipitate was observed), and they were both sterile filtered through a 0.22 μm PVDF filter prior to use in cell culture. Given our ability to verify F10 concentration by Nanodrop as described above, we further confirmed that sterile filtering did not affect the intended F10 treatment concentration. Lupeol (Sigma Aldrich, St. Louis, MO, USA; Merck KGaA, Darmstadt, Germany) solution was made fresh in warm (37 °C) DMSO:Ethanol (60:40) and added directly to wells containing warm culture media supplemented with DMSO:Ethanol solvent. Final concentration of 60:40 DMSO:Ethanol was 0.5% and lupeol was added up to 50 µM final concentration.

MTT Assay. Cells were seeded in 96-well plates at 5 × 10^3^ cells/well and incubated overnight at 37 °C in 5% CO_2_. Each condition was seeded in triplicate. 24 h after seeding, growth media was replaced with fresh media containing the drug solution for 24, 48, or 72 h. Metabolic viability was measured using 3-(4,5-dimethylthiazol-2-yl)-2,5-diphenoyltetrazolium bromide (MTT, 10 µL per well, 5mg/mL) per manufacturer’s protocol (Sigma-Aldrich, St. Louis, MO, USA). After drug treatment, MTT solution was added to media (final concentration = 0.5 mg/mL) and incubated at 37 °C for 4 h. Solubilization solution (10% sodium-dodecyl sulfate containing 0.01 M HCl) was added and incubated at 37 °C overnight. Metabolic viability, measured by absorbance, was recorded on a SpectraMax i3 spectrophotometer plate reader (Molecular Devices, Sunnyvale, CA, USA) at 570 nm, and resulting IC_50_ values were generated using 4 parameter regressions in SoftMax Pro version 6.4 software. MTT assays in were performed independently a minimal of 3 times per cell line.

Nucleic Acid Extraction. Total cellular RNA and DNA were isolated using the illustra TriplePrep Kit per the manufacturer’s protocol (GE Healthcare, Chicago, IL, USA). All nucleic acids were quantified using the NanoDrop 2000C spectrophotometer (Thermo Fisher Scientific, Rockford, IL, USA).

RNA Sequencing. RNA sequencing and analysis was done at the Translational Genomics Core (TGC) at the Stanley S. Scott Cancer Center, LSUHSC, New Orleans, LA, as we have done previously [83]. Briefly, RNA was isolated from cell pellets of untreated cells using the Universal RNA/DNA Isolation kit (Qiagen, Germantown, MD, USA) following the manufacturer’s protocol. Isolated RNA was quantified by using a Qubit (ThermoFisher, Waltham, MA, USA) and its integrity checked on the Agilent Bio Analyzer 2100, G2939A model (Agilent, Santa Clara, CA, USA). Paired-end libraries (2 × 75) were prepared (600 ng per sample) using the TruSeq Stranded mRNA Library Prep kit, validated, and normalized following the recommendations of the manufacturer (Illumina, San Diego, CA, USA). Libraries were sequenced in the NextSeq500 using a High Output Kit v2.5, 150 cycles from Illumina. FASTQ output files were uploaded to Partek Flow, contaminants (rDNA, tRNA, mtrDNA) were removed using Bowtie2 (version 2.2.5) and the reads were aligned to STAR (version 2.5.3a) using the hg19 version of the human genome as reference. Aligned reads were quantified to the hg19-Ensembl Transcript release 75 and normalized by log2 (x + 1) transformation. Normalized counts were used to determine differential gene expression between AA cell lines (SB521 and CHTN06) and CA cell lines (HCT116 and HT29) that served as controls, with gene specific analysis (GSA). All analyses were corrected for multiple comparisons at a false discovery rate (FDR) of 0.05 (at least). Heatmaps were generated using the embedded algorithm in Partek Flow for hierarchical unsupervised comparison of the samples using Euclidian distance. Gene Ontology (GO) enrichment and pathway analysis (with embedded KEGG function) were also done in Partek Flow.

DNA Sequencing. Isolated genomic DNA was PCR amplified. The Ampigene Taq Polymerase kit was used for amplifying each p53 exon. Primers were synthesized by the Stony Brook University Genomics Core Facility using primers previously described by Liu et al. [98]. To validate PCR reactions and relative sizes of the amplicons, PCR products were analyzed using DNA gel electrophoresis. PCR products were purified using the ExosapIt PCR cleanup kit per manufacturer’s instructions. Sanger sequencing of the coding strands was performed by the Stony Brook University Genomics Core. Chromatograms were analyzed from independent duplicate trials depending upon relative initial chromatogram confidence. Mutational status was analyzed using NCBI Blast alignment tool [99] and the reference p53 genome [100] (NCBI: GRCh38.p13, https://www.ncbi.nlm.nih.gov/gene/7157, accessed on 4 June 2020).

Western Blot. Cells were seeded in 100 mm plates at 8 × 10^5^ cells/plate. Cells were treated with fluoropyrimidine concentrations based on 0.5×, 1×, or 2× the IC_50_ value for each cell line/drug condition. The control included the vehicle DMSO or PBS. Cells were washed with ice-cold PBS and lysed with RIPA buffer (Sigma-Aldrich, St. Louis, MO, USA) containing HALT Protease inhibitor cocktail (Sigma-Aldrich, St. Louis, MO, USA). Cell pellets were vortexed every 5 min for 30 min while being kept on ice. Samples were spun at 13,200 RCF at 4 °C for 10 min. Supernatant was saved as isolated protein and cellular debris pellets were discarded. Protein was quantified using the Bradford assay per manufacturer’s protocol (Bio-Rad Laboratories, Inc., Hercules, CA, USA). Protein samples were run on 10% Polyacrylamide Gel Electrophoresis at 100V for 1.5 h and transferred to PVDF membranes (Bio-Rad Laboratories, Inc., Hercules, CA, USA). Primary antibodies were incubated for 2 h at room temperature and included rabbit polyclonal FAS (1:1000, C18C12; Cell Signaling Technology, Danvers, MA, USA), mouse monoclonal CASP8 (1:1000, #9746; Cell Signaling Technology, Danvers, MA, USA), and mouse monoclonal α-Tubulin (1:10,000, #05-829; Millipore, Burlington, MA, USA). Secondary antibodies were incubated for 1 h at room temperature and included mouse anti-rabbit (1:10,000, sc-2357, SantaCruz, Dallas, TX, USA) and horse anti-mouse (1:10,000, #7076S, Cell Signaling Technology, Danvers, MA, USA). The luminol-based enhanced-chemiluminescence autoradiography detection kit (Amersham Biosciences; GE Healthcare, Little Chalfont, UK) was used to assess protein expression. Densitometry was performed using FIJI ImageJ 2.7.0 software and normalized to α-Tubulin signal.

Immunofluorescence. Cells were cultured on 4-well chambered slides (Nunc Lab-Tek, Thermo Fisher Scientific, Waltham, MA, USA) at 25,000 cells/well for 24 h prior to drug treatment. Cells were washed and treated with lupeol and fluoropyrimidine combinations for 72 h, after which the media was removed, and 4% freshly prepared paraformaldehyde was applied for 20 min. Cholera Toxin-B-AlexaFluor-488, for lipid raft staining, (C34775, Thermo Fisher Scientific, Waltham, MA, USA) was prepared at 1 µg/mL in blocking solution and incubated for 30 min prior to 20 min of permeabilization at room temperature. After blocking for 30 min at room temperature, the rabbit FAS primary antibody (same as Western Blot, above) was incubated overnight at 4 °C at 1:120 dilution in blocking buffer. Cy5-anti-rabbit secondary antibody (#102645-330, VWR International, Radnor, PA, USA) was added for 1 h at room temperature at a dilution of 1:100. DAPI (Merck KGaA, Darmstadt, Germany) was added for 5 min at a dilution of 1:4 in IF buffer (PBS with 0.2% Triton X-100 and 0.5% Tween-20). The chamber was removed, and Pro-long Gold Anti-fade Mount (P36930, Thermo Fisher Scientific, Waltham, MA, USA) was used to mount cover slip. Slides were imaged with a Nikon Eclipse 90i epifluorescence microscope.

Lupeol and FAS Receptor Docking Analysis. To assess potential physical interactions between lupeol and the Fas receptor trimer, in silico docking was performed as follows. MedusaDock 2.0 was chosen compared to other platforms (e.g., AutoDock 4.2.6) due to its superior ability to predict binding in induced-fit models of flexible protein structures. Given that the Fas receptor exists in a continuum between its monomeric and trimeric forms [87], MedusaDock 2.0′s induced-fit algorithm was favored over the static/rigid structure algorithm used by other platforms [101]. The ligand (lupeol) 3D structure was downloaded as an .sdf file from the PubChem structure of lupeol and converted to mol2 format [102]. This structure was uploaded to MedusaDock 2.0 with the PDB file ‘2na7’ (the transmembrane domains of the FAS receptor trimer) as the docking site (or ‘receptor’) [87,101,103]. To ensure adequate coverage of the entire trimer structure, two binding sites were set: (1) the first covering the central ~50% of the C3 axis, and (2) the second covering the terminal ~25% of the cytoplasmic and extracellular domains. No amino acid residue constraints were included to limit bias in the docking analysis. The top 2 results were downloaded as .pdb files from MedusaDock 2.0 and visualized in ChimeraX 1.1, with their predicted binding energies (kcal/mol) reported as calculated by the MedusaDock 2.0 algorithm [101,104].

Data Analysis. MTT cell viability regression analysis and IC_50_ value calculation were conducted in Softmax Pro version 6.4 software (Molecular Devices). T-tests, F-tests, and Single-Factor ANOVA were conducted in IBM SPSS version 27 (IBM). DNA sequencing chromatograms were analyzed using SnapGene Viewer 6.0.0 software (SnapGene). Microsoft Excel 365 and Graphpad Prism were used to generate tables and graphs. For all statistical tests, α = 0.05.

## 5. Conclusions

This study comprehensively evaluated the cellular response of racially diverse colorectal cancer (CRC) cell lines to both standard and next-generation fluoropyrimidines. We found that microsatellite instability (MSI) and p53 mutation status were stronger predictors of 5-FU sensitivity than genetic background alone. Transcriptomic analyses uncovered layered gene expression differences driven by both MSI status and racial origin. Despite these extensive differences, including in drug metabolism pathways, we notably demonstrate that F10, a next-generation fluoropyrimidine polymer, exhibits superior potency and consistency compared to 5-FU across all tested cell lines, including those with traditionally resistant profiles to 5-FU. This finding is strengthened by the inclusion of African American (AA) patient-derived CRC cell lines, given clinical trends of differential response to fluoropyrimidine-based therapies. The improved efficacy of F10 vs. 5-FU was independent of race, MSI, and p53 status, suggesting that F10 overcame conventional resistance mechanisms. Our work further adds to the characterization of the antitumor effects of the dietary micronutrient lupeol and offers a novel therapeutic strategy in combining it with F10. Therefore, lupeol’s role as a dietary adjuvant warrants deeper exploration through pathway-specific assays. To definitively predict systemic toxicity and therapeutic potential of these agents, investigation of F10 in preclinical models (organoids and in vivo systems) is necessary. Though the limited availability of the F10 chemotherapeutic agent initially prevented us from including such ex vivo/in vivo experiments in the present study, this pre-clinical work is in the process of being initiated. Overall, our present findings contribute to the development of more inclusive, biomarker-informed CRC treatments and highlight the value of integrating diverse tumor models and dietary adjuvants into chemotherapeutic research.

## Data Availability

The raw data (FASTQ) generated for this study can be found in the Gene Expression Omnibus (GEO) with the accession number GSE146009.
